# Update on Dosing and Fractionation for Neoadjuvant Radiotherapy for Localized Soft Tissue Sarcoma

**DOI:** 10.1007/s11864-024-01188-2

**Published:** 2024-03-13

**Authors:** Siyer Roohani, Lisette M. Wiltink, David Kaul, Mateusz Jacek Spałek, Rick L. Haas

**Affiliations:** 1 Department of Radiation Oncology, Charité − Universitätsmedizin Berlin, corporate Member of Freie Universität Berlin and Humboldt-Universität zu Berlin, Augustenburger Platz 1, 13353 Berlin, Germany; 2 BIH Charité Junior Clinician Scientist Program, BIH Biomedical Innovation Academy, Berlin Institute of Health at Charité − Universitätsmedizin Berlin, Charitéplatz 1, 10117 Berlin, Germany; 3 German Cancer Consortium (DKTK), Partner site Berlin, and German Cancer Research Center (DKFZ), Heidelberg, Germany; 4 Department of Radiation Oncology, Leiden University Medical Center, Leiden, the Netherlands; 5 Department of Soft Tissue/Bone Sarcoma and Melanoma, Maria Sklodowska-Curie National Research Institute of Oncology, Warsaw, Poland; 6 Department of Radiotherapy I, Maria Sklodowska-Curie National Research Institute of Oncology, Warsaw, Poland; 7 Department of Radiation Oncology, The Netherlands Cancer Institute, Amsterdam, the Netherlands

**Keywords:** Soft tissue sarcoma, Neoadjuvant radiotherapy, Preoperative radiotherapy, Retroperitoneal, Extremity, Hypofractionation

## Abstract

Neoadjuvant radiotherapy (RT) over 5–6 weeks with daily doses of 1.8–2.0 Gy to a total dose of 50–50.4 Gy is standard of care for localized high-grade soft tissue sarcomas (STS) of the extremities and trunk wall. One exception is myxoid liposarcomas where the phase II DOREMY trial applying a preoperative dose of 36 Gy in 2 Gy fractions (3–4 weeks treatment) has achieved excellent local control rates of 100% after a median follow-up of 25 months.

Hypofractionated preoperative RT has been investigated in a number of phase II single-arm studies suggesting that daily doses of 2.75–8 Gy over 1–3 weeks can achieve similar oncological outcomes to conventional neoadjuvant RT. Prospective data with direct head-to-head comparison to conventional neoadjuvant RT investigating oncological outcomes and toxicity profiles is eagerly awaited.

For the entire group of retroperitoneal sarcomas, RT is not the standard of care. The randomized multi-center STRASS trial did not find a benefit in abdominal recurrence-free survival by the addition of preoperative RT. However, for the largest histological subgroup of well-differentiated and grades I and II dedifferentiated liposarcomas, the STRASS trial and the post-hoc propensity-matched STREXIT analysis have identified a possible benefit in survival by preoperative RT. These patients deserve to be informed about the pros and cons of preoperative RT while the longer follow-up data from the STRASS trial is awaited.

## Introduction

Perioperative radiotherapy (RT) improves local control in patients with high-grade soft tissue sarcoma (STS) of the extremities and trunk wall [1–3]. Although the oncological outcomes of pre- vs. postoperative RT are similar, preoperative RT leads to a more acute yet reversible toxicity compared to postoperative RT causing more late and irreversible side effects such as fibrosis, joint stiffness, and lymphedema [4, 5]. Therefore, preoperative RT is now the preferred therapy sequence for high-grade STS of the extremities and trunk wall [6, 7]. The conventional fractionation of 1.8–2 Gy per day for 5–6 weeks to a total dose of 50–50.4 Gy used in previous clinical trials is now being challenged by numerous phase II single-arm studies suggesting equivalent clinical outcomes with single doses of 2.75–8 Gy delivered in 1–3 weeks [8•].

For the entire group of retroperitoneal STS as such, perioperative RT is not considered standard of care [6, 9]. However, for the largest histological subgroup of well-differentiated and grades I and II dedifferentiated liposarcomas, the prospective multi-center randomized phase III (EORTC-62092) STRASS trial from the European Organization for Research and Treatment of Cancer—Soft Tissue and Bone Sarcoma Group (EORTC-STBSG) and the post-hoc propensity-matched STREXIT analysis have identified a possible benefit in abdominal recurrence-free survival by preoperative RT [10••, 11••]. If perioperative RT is considered as part of the treatment for retroperitoneal STS, preoperative RT is strongly recommended [6].

This review gives an update on the dosing, target volume definition, fractionation, and the role of neoadjuvant RT for localized STS of the extremities, trunk wall, and retroperitoneal STS.

## Extremity and trunk wall STS

### Dosing and fractionation

Neoadjuvant RT for intermediate and high-grade STS of the extremity and trunk wall is conventionally delivered in 1.8–2.0 Gy daily fractions over a total treatment time of 5–6 weeks to a total dose of 50–50.4 Gy [6]. One exception where dose de-escalation is possible is myxoid liposarcomas particularly known for their radiosensitivity where the phase II DOREMY trial applying a preoperative dose of 36 Gy in 2 Gy fractions (3–4 weeks treatment) has achieved excellent local control rates of 100% after a median follow-up of 25 months [12•, 13].

### Target volume delineation

The target volume is delineated on T1-weighted, post-gadolinium magnetic resonance imaging (MRI) scans registered with planning computed tomography (CT) scans [6, 14]. The gross tumor volume (GTV) encompasses the visible tumor volume. For deeply seated STS below or reaching the fascia, the GTV is expanded by safety margins of 1.5 cm radially and 3–4 cm longitudinally anatomically constrained along the muscles, where microscopic tumor spread is expected, e.g., in the peritumoral edema and biopsy tracts (whenever visible), to obtain the clinical target volume (CTV) [6, 14]. For subcutaneous STS not involving the fascia, the GTV is expanded 3–4 cm circumferentially and 0.5–1 cm into underlying non-involved muscle while including peritumoral edema and biopsy tracts (whenever visible) to form the CTV [6]. To adjust for movements or inaccuracies during the daily positioning of patients, an additional margin of 5 mm is added to the CTV to form the planning target volume (PTV) if daily image guidance is applied and 1 cm or more if not. When relapses occur, they are most often found inside the target volume, thereby supporting the need for these comparatively large safety margins [15–17].

### Hypofractionation

Hypofractionated RT is conventionally regarded as irradiation with single daily doses of more than 2.2 Gy, although there is no clear definition. There are two rationales for hypofractionated RT. From a radiobiological perspective, some tumor entities with low α/β ratios such as prostate and breast cancer are more effectively eradicated with hypofractionated RT [18, 19]. For STS, the α/β ratio varies between the subtypes but is also comparably low overall ranging from 4–5 Gy and thus provides a radiobiological rationale for hypofractionation [20]. Another argument for hypofractionation is patient convenience, as conventional courses of several weeks can be substantially shortened to a smaller number of RT sessions particularly useful for older, immobile, and frail patients [21]. Depending on the country and insurance reimbursement system, hypofractionation can also be of economic interest as shorter RT courses reduce costs per patient for the healthcare system and allow a higher patient throughput which may be especially important in low-income regions where access to radiation oncology facilities may be limited [22, 23]. The possibility to treat more patients in reference tumor centers is particularly important in the management of sarcomas where treatment in experienced high-volume centers has repeatedly shown a survival advantage [24, 25]. Importantly, the common 1.8–2.0 Gy fractions per day for STS do not stem from strong clinical or radiobiological data but were rather applied by previous conventions. Nevertheless, the increased risk of both early and late toxicity counteracts the aforementioned advantages and arguments for hypofractionation [26]. There is a large amount of robust prospective clinical evidence for hypofractionation in prostate, breast, and rectal cancers where it has proven at least non-inferior to conventionally fractionated RT in oncological outcomes and toxicity profiles and has therefore become standard of care [27–32].

Over the last decade, a number of systematic review manuscripts concerning clinical studies, investigating neoadjuvant hypofractionated RT, demonstrated similar local control with toxicity profiles comparable to conventionally fractionated RT in extremity and trunk wall STS [33–35]. Most studies commonly compare their outcomes and define their toxicity endpoints according to the landmark CAN-NCIC-SR-2 trial where preoperative RT with 50 Gy in 2 Gy fractions resulted in acute toxicity (major wound complications within 120 days after surgery as defined by the authors) in 34% of patients and local control of 93% after 5 years, although the study was powered for the acute toxicity endpoint only [4, 5].

A moderately hypofractionated dosing regimen of preoperative 15 × 2.85 Gy, corresponding to an equivalent dose in 2 Gy fractions (EQD2) of 48.8 Gy assuming the aforementioned α/β value of 4 Gy, delivered over 3 weeks by Guadagnolo et al. in 120 patients led to 37% major wound complications comparable to the SR-2 trial [4, 20, 36]. The last patient was enrolled on January 6th, 2021, and oncological endpoints with longer follow-ups are awaited with interest.

In all the references that are cited here below, 5-day schedules are reported, yet with a gradual increase of the dose per fraction from 5 Gy up to 8 Gy.

Koseła-Paterczyk used a more hypofractionated regimen of preoperative 5 × 5 Gy regimen delivered in 5 consecutive days to 311 patients. This regimen was associated with a major wound complication rate of 24% and achieved local control rates of 86.2% after a median follow-up of 57 months [37, 38]. While the low acute toxicity rate is promising; the local control rate of 86.2% compared to the 93% 5-year local control in the SR-2 study is a cause for concern. Underlying reasons may be, first, the use of older three-dimensional conformal radiation therapy techniques which appeared inferior in local tumor control to intensity-modulated radiation therapy in large retrospective cohort studies [37, 39, 40]. And second, biologically, 5 × 5 Gy corresponds to an EQD2 of 37.5 Gy which is substantially lower than the accepted 50 Gy regimen of the SR-2 study [20].

Gobo Silva et al. [41] also used 5 × 5 Gy and combined it with concomitant doxorubicin and ifosfamide in 18 patients and detected 33% major wound complications and one local recurrence after a median follow-up of 29 months (94% local control). Although the sample size is too small and the longer follow-up pending to assess the local disease control, the 33% major wound complication rates within 120 days are again suggesting non-inferior acute toxicities to conventional RT dosing.

Myxoid liposarcomas again stand out with their high radiosensitivity in the study of Koseła-Paterczyk et al. who achieved a 100% local control rate after a median follow-up of 27 months with preoperative 5 × 5 Gy (EQD2: 37.5 Gy) comparable to the 100% local control after 36 Gy conventional fractionation in the DOREMY study after a median follow-up of 25 months [12•, 42]. Due to this extraordinary radiosensitivity, future trials investigating alternative RT regimens should consider excluding myxoid liposarcomas to prevent bias and focus on the more common and difficult-to-treat STS subtypes.

Kalbasi et al. [43] applied an even more hypofractionated regimen of 5 × 6 Gy (EQD2: 50 Gy) biologic equivalent to the normofractionated regimen of 50 Gy in 50 patients. Major wound complication rates of 32% remained comparable with the SR-2 data and long-term local control data is pending. The study again emphasizes the practicality of short RT regimens by an increase in patient accrual and patients traveling to the sarcoma reference center noted by the authors compared to the conventional 2–50 Gy fractionation before study initiation [43].

Bedi et al. [44] treated 32 patients with a further hypofractionated preoperative RT regimen of 5 × 7 Gy (EQD2: 64.2 Gy) delivered every other day over 2 weeks and found a 25% major wound complication rate, 91% clear surgical margins and a remarkable 100% local control rate after a median follow-up of 36.4 months for which the study was powered. A minority of 31.5% of participating patients received 3 cycles of concomitant doxorubicin/ifosfamide. Again, even with the 5 × 7 Gy, the acute toxicity remains favorable. Reasons for the low toxicity may be the smaller safety margins of 2 cm longitudinally (instead of 3–4 cm) and 1 cm radially (instead of 1.5 cm) and the daily cone beam CT image guidance allowing for a smaller PTV margin of 5 mm, which was previously shown to reduce toxicity compared to the CAN-NCIC-SR-2 trial where image-guided radiation therapy was not yet applied [45].

Longer follow-up data with the 5 × 7 Gy image-guided radiotherapy regimen every other day were published by Kubicek et al. [46]. In 16 patients (15 with 5 × 7 Gy, 1 with 5 × 8 Gy) the authors reported 3 patients (20%) with wound complications, 1 patient (6.7%) with late grade 4 contracture requiring surgery, and 1 (6.7%) in-field local recurrence 100 days after resection with positive surgical margins [46]. Although the sample size is small, also this regimen appears feasible, well-tolerated, and in no studied parameter inferior to previous data on conventional fractionation.

Leite et al. [47] reported outcomes on the most hypofractionated treatment regimen in the literature to date. In 25 patients, 5 × 8 Gy (EQD2: 80 Gy) image-guided neoadjuvant radiotherapy was given every other day. Formally, the study met its primary endpoint by detecting major wound complication rates of 28%. However, 3 patients underwent vascular occlusions in the irradiated area eventually leading to amputations, and 1 patient with grade 3 motion dysfunction received amputation as well. After thorough analysis, the authors found the areas of vascular occlusion not to have received a “significant dose”, assuming that the high doses may have caused indirect vascular effects affecting the surgical outcome [47]. Regardless of the underlying pathophysiology and although these striking toxicities do not cover the CAN-NCIC-SR-2 trial definition of major wound complication, to our opinion, the toxicities are certainly displaying a too aggressive therapy regimen despite comparable local control rates. Radiobiologically, the findings demonstrate how a “seemingly small” difference of 5 × 8 Gy (EQD2: 80 Gy) compared to 5 × 7 Gy (EQD2: 64.2 Gy) in the studies by Bedi et al. and Kubicek et al. can sharply raise biological tissue damage in late responding normal tissue in STS patients [44, 46].

Remarkably, however, the pre-treatment median tumor size of 14 cm in the study by Leite et al. [47] was reduced by preoperative RT with 5 × 8 Gy every other day to 10.5 cm in the reassessment MRI before surgery, a reduction of 25%. Notably, only 3 cases were myxoid liposarcomas. Furthermore, 96% of tumors were resected with clear surgical margins. This observation is clearly in contradiction to data after conventionally fractionated RT where radiological responses are rarely seen [48, 49]. Moreover, this observation might challenge the previous notion that preoperative RT cannot improve resectability [50].

Currently, there is no published data available on a direct head-to-head comparison of hypofractionated RT to conventionally fractionated RT. To the best of our knowledge, the only clinical trial with such a randomization is the NCT04425967 of which the results are eagerly awaited.

To conclude this paragraph, surgery remains the mainstay of curative limb-sparing therapy, and clear surgical margins are an important risk factor for local disease recurrence and subsequent morbidity [51–53]. Although the addition of RT, preferably before surgery, is able to increase the rate of local control, it comes at the cost of both acute and late toxicity profiles. These profiles are dependent upon the total dose applied and very likely even more on the fraction size by which the total dose is delivered.

## Retroperitoneal STS

### Patient selection

In contrast to STS arising in the extremities, neoadjuvant RT is not the standard of care for an unselected group of STS of the retroperitoneum [6, 9, 54]. The only comparative phase III data is derived from the multi-center STRASS trial (EORTC-62092) comparing neoadjuvant RT and surgery to surgery alone and did not find a significant difference in abdominal recurrence-free survival after a median follow-up of 43.1 months across all histological subtypes taken together [10••]. However, unplanned subgroup analysis and the post-hoc propensity score-matched STREXIT analysis, matching enrolled STRASS study patients to not enrolled patients from the same study centers, have proposed a potential benefit in abdominal recurrence-free survival by preoperative RT for primary well-differentiated liposarcomas (WDLPS) and grades 1 and 2 dedifferentiated liposarcomas (DDLPS) [10••, 11••]. Per protocol, STRASS will be reanalyzed with a longer follow-up of which the results are awaited.

Moreover, a pattern of recurrence analysis in over 1000 retroperitoneal STS cases from eight high-volume centers, investigating the most common histological subtypes of retroperitoneal STS, suggests that WDLPS rarely if ever metastasize, yet continuously relapse locally in a non-plateauing fashion even after 6 years of follow-up [55]. Primary DDLPS, the most common subtype, has a similar, non-plateauing, local recurrence pattern but with a much steeper incline while also showing distant recurrences in a smaller proportion of cases. Within the DDLPS group, grades I and II tumors display a local recurrence pattern almost similar to WDLPS, while grade III tumors relapse both locally and distantly, thereby exhibiting the highest mortality of all histological subtypes. For leiomyosarcomas, the risk of distant metastases (up to 50% after only 4 years of follow-up) is substantially higher than the risk of local recurrence [55].

These very informative data suggest (i) WDLPS are predominantly, and DDLPS in the majority of cases, a topic of local management and require aggressive combined local therapies to improve oncological outcomes; (ii) longer follow-up is needed to observe a possible benefit of combined surgery and RT for WDLPS and DDLPS over surgery alone as this may become apparent only after prolonged follow-up; (iii) potentially, leiomyosarcomas and grade III DDLPS may benefit from systemic management alongside surgery, and in order to test this hypothesis, STRASS-2 is currently accruing these 2 patient cohorts (NCT04031677).

To conclude this paragraph, although the STRASS trial is currently published as a negative study, highly selected retroperitoneal liposarcoma patients might benefit from preoperative RT. These patients deserve to be informed about the pros and cons of preoperative RT while the longer follow-up data from the STRASS trial is awaited. There is insufficient data to offer routine preoperative RT to patients with other histological subtypes.

### Dosing and fractionation

Currently, if applied, a dose of 1.8–2.0 Gy once daily to a total dose of 50–50.4 Gy in 5–6 weeks is recommended [6, 9]. No prospective clinical data exist for hypofractionation which may be owed to the tumor’s proximity to radiosensitive structures in the abdomen; as the posterior wall is regarded as a high-risk region for positive surgical margins and local tumor recurrence, the concept of preoperative RT dose escalation to this anatomical area has been investigated [56, 57]. The first phase I and early phase II experience (median follow-up 23 months) escalating the dose to 63 Gy relative to the biological effective dose in 28 fractions of proton beam radiation to a 2–0.5-cm region in the posterior wall showed promising local control rates with acceptable toxicity [58, 59]. More recently, the largest single-center retrospective cohort study using a median dose escalation to 57.5 Gy to a similar 2–2.5-cm-thick region in the posterior wall suggested a substantial benefit in local abdominal tumor control and recurrence-free survival [60].

### Target volume delineation

The GTV encompasses the visible tumor volume and is delineated on contrast-enhanced CT images [6, 9]. For tumors located above the pelvic brim, breathing motion adjustments with 4D-CTs are recommended. In this case, the GTV is contoured in all phases of breathing and merged together to obtain the internal GTV (iGTV). For tumors above the pelvic brim, the iGTV is expanded by 1.5 cm isotropically and anatomically constrained at bony and bowel loop surfaces to create the internal target volume (ITV), and for tumors below the pelvic brim, the GTV is expanded by 1.5 cm and also anatomically constrained to create the CTV. The manual adaption to adjacent anatomical structures serves to omit macroscopically non-infiltrated normal tissue structures such as bowel loops, kidneys, skin surface, bones, and liver. A safety distance to such structures should be maintained such as 5 mm to bowel loops and 3–5 mm to skin surface. Consultation with the treating surgeon is recommended so that, for example, the ipsilateral kidney does not need to be spared from incidental radiation dose, if a nephrectomy is planned. In case of tumor extension from the retroperitoneum to the inguinal canal without scrotal involvement, an additional 3 cm inferior expansion is recommended. The PTV margin added to the ITV or CTV is institute-specific and dependent on the daily reproducibility of patient positioning. In the case of daily image guidance, a margin of 5 mm might suffice, while larger margins of 9–12 mm are advised when image guidance is not carried out. In contrast to extremity and trunk wall STS, the data on the pattern of recurrence for retroperitoneal STS after preoperative RT and surgery with respect to RT volumes is very limited [56]. Thus, the aforementioned safety margins are rather based on conventional experts’ consensus than on empirical data.

## Conclusions and future perspectives

For extremity and trunk wall STS, preoperative RT followed by wide surgical excision remains the standard of care as it achieves high local tumor control with acceptable toxicity. To increase patient convenience and perhaps improve resectability, hypofractionation is being increasingly investigated in prospective clinical trials exploring various dosing schedules, with promising data thus far. However, outside the setting of such trials, or even prospective registries, hypofractionation should still be considered as experimental and performed only in high-volume tertiary sarcoma centers.

For retroperitoneal STS, the STRASS trial has shown that studying a subtype agnostic, heterogenous group of tumors is unwise and subsequently and consequently showed no benefit of preoperative RT for the RPS patient population as a whole. Longer follow-up data is needed to more reliably investigate the impact of preoperative RT for WDLPS and grades 1 and 2 DDLPS. To generate new hypotheses regarding RT dose-escalation or de-escalation of therapies overall, a detailed, subtype- and treatment-specific pattern of recurrence analysis from the STRASS data is needed.

## Data Availability

No datasets were generated or analysed during the current study.

## Abbreviations

CT: Computed tomography
CTV: Clinical target volume
DDLPS: Dedifferentiated liposarcomas
EORTC: European Organization for Research and Treatment of Cancer
EQD2: Equivalent dose in 2 Gy fractions
GTV: Gross tumor volume
iGTV: Internal gross tumor volume
MRI: Magnetic resonance imaging
PTV: Planning target volume
RT: Radiotherapy
STS: Soft tissue sarcoma
WDLPS: Well-differentiated liposarcomas
